# Cytosolic Phospholipase A2 Regulates TNF-Induced Production of Joint Destructive Effectors in Synoviocytes

**DOI:** 10.1371/journal.pone.0083555

**Published:** 2013-12-12

**Authors:** Randi M. Sommerfelt, Astrid J. Feuerherm, Kymry Jones, Berit Johansen

**Affiliations:** Department of Biology, Norwegian University of Science and Technology, Trondheim, Norway; Chang Gung University, Taiwan

## Abstract

**Introduction:**

Rheumatoid arthritis (RA) is an inflammatory disease of the joint characterized by chronic synovitis causing pain, swelling and loss of function due to destruction of cartilage and bone. The complex series of pathological events occurring in RA is largely regulated via excessive production of pro-inflammatory cytokines, the most prominent being tumor necrosis factor (TNF). The objective of this work was to elucidate possible involvement of group IVA cytosolic phospholipase A2 (cPLA2α) in TNF-induced regulation of synovitis and joint destructive effectors in RA, to evaluate the potential of cPLA2α as a future therapeutic target.

**Methods:**

The involvement of cPLA2α in tumor necrosis factor (TNF)-induced intracellular signaling cascades in synoviocytes (synovial fibroblast-like cells) was analyzed by arachidonic acid (AA) release assay, synoviocyte enzyme activity assay, gene expression analysis by real-time PCR and ELISA immunoassay for the detection of prostaglandin E2 (PGE2), interleukin 8 (IL8) and stromelysin-1 (MMP3), respectively.

**Results:**

Inhibitors of cPLA2α enzyme activity (AVX002, ATK) significantly reduced TNF-induced cellular release of AA, PGE2, IL8 and MMP3. This reduction was evident both at transcriptional, protein or metabolite levels. Interestingly, cPLA2α inhibition affected several key points of the arachidonyl cascade; AA-release, cyclooxygenase-2 (COX2) expression and PGE2 production. Furthermore, the results suggest that cPLA2α is subject to transcriptional auto-regulation as inhibition of cPLA2α resulted in reduced PLA2G4A gene expression in TNF-stimulated synoviocytes.

**Conclusions:**

cPLA2α appears to be an important regulator of central effectors of inflammation and joint destruction, namely MMP3, IL8, COX2, and PGE2. Decreased transcription of the PLA2G4A and COX2 genes in response to cPLA2α enzyme inhibition further suggest a self-reinforcing effect of cPLA2α inhibition in response to TNF. Collectively, these results support that cPLA2α is an attractive therapeutic target candidate as its inhibition reduces the production of multiple key pro-inflammatory factors involved in RA pathogenesis.

## Introduction

Rheumatoid arthritis (RA) is an auto-immune and systemic inflammatory disease affecting 0.5-1% of the population, worldwide. In RA, chronic synovitis causes pain, swelling and loss of joint function due to degradation of cartilage and bone erosion [1]. Activated fibroblast-like synoviocytes (FLS) in the inflamed synovium are important contributors to arthritis through supranormal production of prostanoids, cytokines, chemokines, matrix degrading enzymes, angiogenic factors and adhesion molecules, thus perpetuating inflammation and joint destruction [2]. A key mechanism in the destructive signaling loop of RA is a dysregulation of the level of the pro-inflammatory cytokine tumor necrosis factor (TNF) [3,4]. TNF is overexpressed in RA synovium where it elicits a variety of biological effects on inflammation and immunity including modulation of gene expression and inflammatory joint destruction [5]. 

Phospholipase A2 (PLA2) enzymes release unsaturated fatty acids such as arachidonic acid (AA) by hydrolysis of the *sn*-2 ester bond of membrane glycerophospholipids. The arachidonyl specific group IVA cytosolic PLA2 enzyme (cPLA2α) encoded by the PLA2G4A gene is a major contributor to the elevated levels of AA in inflammation [6,7]. cPLA2α activity is regulated at many levels; by increased intracellular Ca^2+^ levels in response to pro-inflammatory stimuli, by binding to lipid second messengers, by phosphorylation induced by kinases, and by *de novo* gene transcription [8–10]. Following cPLA2α activation, the released AA is enzymatically metabolized to bioactive eicosanoids including prostaglandins, thromboxanes, lipoxins and leukotrienes [11]. Prostaglandin E2 (PGE2) is synthesized from AA through the cyclooxygenase (COX) pathway and is generally recognized as a potent lipid regulator of active inflammation [12]. The beneficial anti-inflammatory effect of reducing PGE2 synthesis is well recognized, and as such, non-steroidal anti-inflammatory drugs (NSAIDS) targeting the COX enzymes are widely used for symptomatic relief in RA [13]. However, long term use of NSAIDS has adverse effects e.g. affecting the gastrointestinal- and cardiovascular system and bone homeostasis [14–16]. The development of TNF-blocking agents has revolutionized the treatment of RA-patients and TNF-blockers are frequently used in RA therapy. However, approximately one-third of patients do not respond successfully to treatment [17]. Anti-TNF therapies are also under scrutiny following reports of malignancies, serious infections and long-term safety concerns [18,19]. Therefore, a search for alternative therapeutic targets is of great interest. 

Several lines of evidence point to a role for cPLA2α in arthritis and inflammation, although the exact mechanisms of how cPLA2α regulates disease activity is not fully elucidated [7,20–23]. The aim of this study was to investigate the involvement of cPLA2α in joint and bone-destructive signaling in human synoviocytes. We identified cPLA2α as a regulator of TNF-induced expression of key players in RA pathology involved in bone and cartilage destruction, angiogenesis and neutrophil recruitment, namely stromelysin-1 (matrix metalloproteinase 3, MMP3), interleukin 8 (IL8), COX2 and PGE2. Furthermore, our results suggest that cPLA2α is subject to auto-regulation as inhibition of cPLA2α activity leads to reduced expression of PLA2G4A mRNA in response to TNF. Hence, our results support the comprehension that cPLA2α may be a major contributor to synovitis and joint destruction in RA, and therefore a potent therapeutic target candidate. 

## Materials and Methods

### Reagents

Recombinant human TNF was from R&D systems (Abingdon, UK). Arachidonyl trifluoromethyl ketone (AACOCF3, ATK) was from Enzo Life Sciences (Farmingdale, NY, USA). PBS was from Oxoid (Basingstoke, Hampshire, UK). [^3^H]-arachidonic acid ([^3^H]-AA), and liquid scintillation cocktail Ultima Gold were from NEN Perkin Elmer (St. Louis, MO, USA). Leupeptin and pepstatin were from Roche Molecular Biochemicals (Indianapolis, USA). M-MLV reverse transcriptase, dNTPs and DTT were from Invitrogen (St. Louis, MO, USA). Random hexamer primers and RNAsin were from Promega (Madison, WI, USA). DNAse- and RNAse-free water was from VWR (Pennsylvania, USA). RNeasy® minikit was from Qiagen (Valencia, CA, USA), ELISA kits for PGE2, IL8 and MMP3 were from Cayman Chemicals (Ann Arbor, MI, USA), Bender Medsystems (Vienna, Austria) and RayBiotech (Norcross, GA, USA), respectively. AVX002 was provided by Avexxin AS (Trondheim, Norway) and synthesized by Synthetica AS (Oslo, Norway). All other reagents were from Sigma-Aldrich (St. Louis, MO, USA).

### Cell Culture

The human synovial sarcoma derived cell line SW982 was purchased from ATCC (London, UK). The cells were maintained in Dulbecco's Modified Eagle Medium (DMEM) supplemented with 10% FBS, 0.1 mg/mL gentamicin and 0.3 mg/mL L-glutamine at 37°C with 10% CO_2_. Experiments were performed at 3 days post-confluence following overnight serum deprivation in serum-free DMEM. When inhibitors were applied, cells were pretreated for 2 hrs before stimulation with TNF (10 ng/mL).

### [^3^H]-arachidonic acid release assay

Cells were labeled for 18 hrs with [^3^H]-AA (0.4 μCi/mL) in serum-free DMEM before experimental treatment. [^3^H]-AA release was analyzed in triplicates as previously described [24]. The results shown are released [^3^H]-AA in supernatants relative to total [^3^H]-AA incorporated into the cells. IC50 values for inhibitors were calculated as mean ± SD of at least 3 independent experiments.

### Assay of cellular cPLA2α enzyme activity

SW982 synoviocytes were serum starved over-night before stimulation with TNF (10 ng/mL, 6 hours). Cells were lysed and 200 μg of total protein was analyzed for cPLA2α activity as described [24–27]. Bromoenol lactone (25 μM) and dithiothreitol (2.36 mM) were included in all reactions to inhibit activity of iPLA2 and sPLA2 [27].

### Real-time reverse-transcription polymerase chain reaction (RT^2^-PCR)

Total RNA was isolated using RNeasy® minikit (Qiagen) according to kit protocol. RNA concentrations and integrity was monitored by NanoDrop spectrophotometric measurement (NanoDrop Technologies Inc. Wilmington DE, USA) and total RNA (1 µg) was reverse transcribed as described in [24]. Specific primers for IL8, MMP3, COX2, PLA2G4A and GAPDH with the following sense and antisense primers, were used in standard real-time RT-PCRs, with SYBR Green as fluorescence reporter: IL8, 5´-GACATACTCCAAACCTTTCCAC-3´ and 3´-CTTCTCCACAACCCTCTGC-5´, MMP3, 5´-TGATGAACAATGGACAAAGGATAC -3´ and 3´-CTGTGAGTGAGTGATAGAGTGG-5´, COX2, 5´-GGGGATCAGGGATGAACTTT -3´ and 3´-TGGCTACAAAAGCTGGGAAG -5´, PLA2G4A, 5´-CATGCCCAGACCTACGATTT -3´ and 3´-CCCAATATGGCTACCACAGG -5´, GAPDH, 5´-CATCAAGAAGGTGGTGAAGCAG-3´ and 3´-TGTAGCCAAATTCGTTGTCATACC-5´. Cq values for each amplification curve were calculated by the Mx3000P software (Stratagene). Fold changes in mRNA expression and statistical analysis were calculated by the REST 2009 software [28] with mean PCR reaction efficiencies calculated by the LinRegPCR software [29] using GAPDH as reference gene.

### Enzyme-linked immunosorbent assay (ELISA)

ELISA analyses were performed according to their respective kit protocols. The read-out for all ELISAs was carried out with a Multiscan plate reader (Ascent Labsystems). The corresponding Ascent software for Multiscan, Version 2.4.1 was used to obtain the data. Mean estimated IC50 value for PGE2 production was calculated from 3 independent experiments.

### Statistical analysis

For AA release and ELISA analysis, statistical analyses were performed in SPSS Statistics 20 using one-way ANOVA at 95% confidence level in conjunction with Tukey HSD test. For real-time PCR data, statistical analysis was performed by the REST 2009 software [28]. Differences were considered significant at p ≤ 0.05. 

## Results

### TNF is a potent inducer of joint destructive regulators in synoviocytes

**Figure 1:**
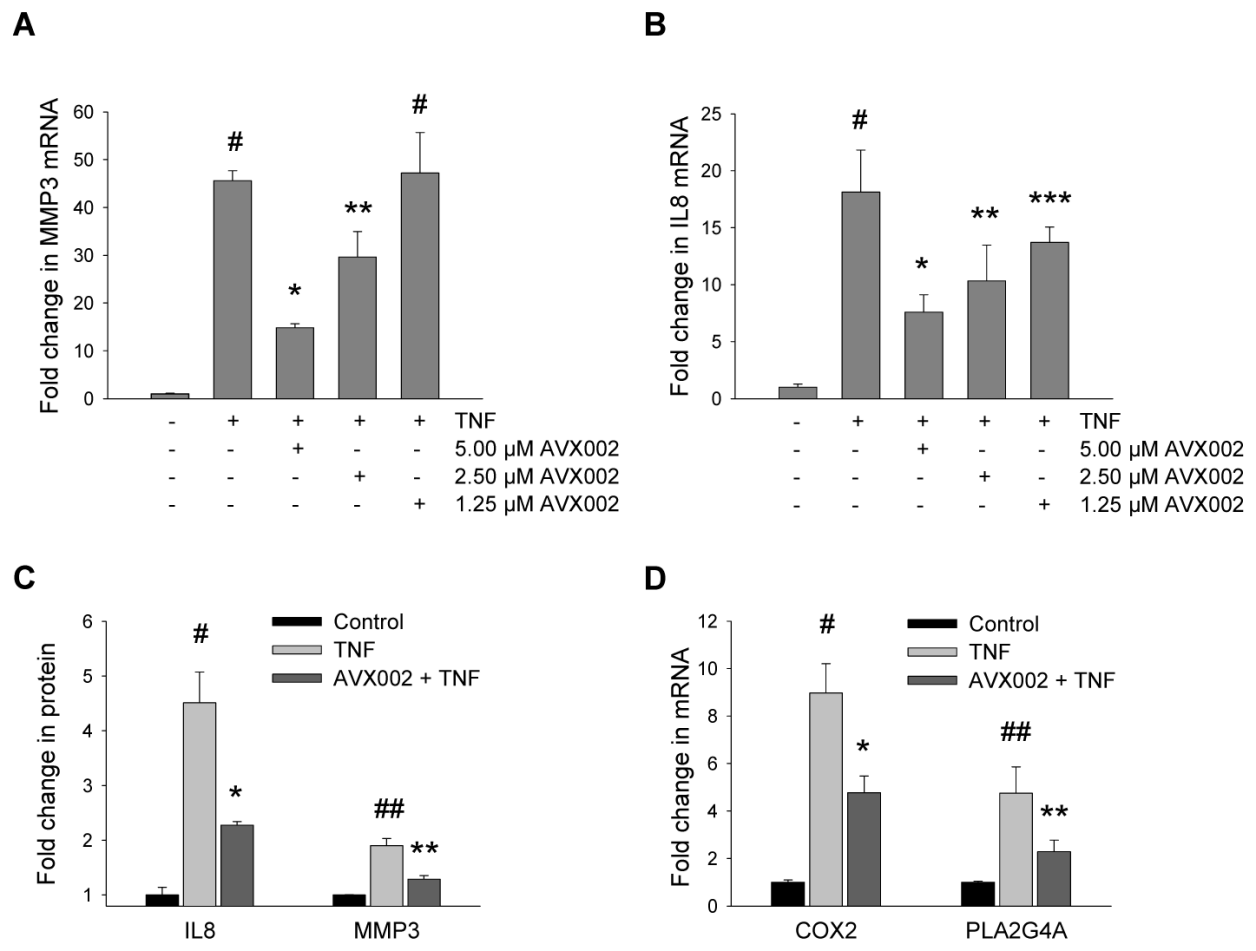
Inhibition of cPLA2α reduces TNF-induced expression of PLA2G4A, COX2, MMP3 and IL8. Fibroblast-like synoviocytes were treated with AVX002 (2 hrs) in indicated concentrations (A, B), 10 µM (C), or 5 µM (D), prior to TNF stimulation (10 ng/mL, 24 hrs). Total RNA was isolated and transcription of MMP3 (A) and IL8 (B), PLA2G4A and COX2 (D), was analyzed by real-time PCR as described in the Methods section. Amplification efficiency of all primer pairs were calculated by the LinRegPCR software and fold-change in gene expression compared to untreated samples was calculated by the REST 2009 software with GAPDH as reference gene. Supernatants were collected and analyzed by ELISA for MMP3 and IL8 protein (C) as described in the Method section (note starting point of Y-axis at 0.8). Data shown in all graphs are mean ± SEM (A, B, D) or mean ± SD (C) fold change compared to untreated samples for one representative of at least three independent experiments performed in duplicates. Significance is indicated as follows: A) ^#^p ≤ 0.01 vs control; ^*^p, ^**^p ≤ 0.01 vs control and TNF-treated cells. B) ^#^p ≤ 0.01 vs control; ^*^p, ^**^p, ^***^p ≤ 0.03 vs control and TNF-treated cells. C) IL8: ^#^p ≤ 0.01 vs control; ^*^p ≤ 0.01 vs control and TNF-treated cells. MMP3: ^##^p ≤ 0.01 vs control, ^**^p ≤ 0.01 vs control and TNF-treated cells. D) COX2: ^#^p ≤ 0.01 vs control; ^*^p ≤ 0.03 vs control and TNF-treated cells. PLA2G4A: ^##^p ≤ 0.02 vs control; ^**^p ≤ 0.03 vs TNF-treated cells.

MMP3, IL8 and PGE2 are important regulators of inflammation and joint destruction in RA [12,30,31]. Matrix metalloproteinases (MMPs) are main contributors to RA cartilage destruction and the levels of several MMP subgroups, including MMP3, are elevated in RA synovial fluid [30,32]. The chemokine IL8 is overexpressed in RA synovium and acts as an angiogenic factor and chemoattractant for neutrophils thereby maintaining persistent migration of inflammatory cells into the synovium [31,33], while PGE2 is a powerful inducer of inflammation [12]. As TNF is known to induce both IL8 and MMP3 [3,34], we first characterized basal and TNF-induced gene expression by real-time PCR and protein by ELISA to justify the use of SW982 synoviocytes as a model for studying the TNF response. Indeed, TNF increased mRNA expression of MMP3 and IL8 by 45.6 ± 2.1-fold (p ≤ 0.01), and 18.1 ± 3.7-fold (p ≤ 0.01), respectively (Figure 1A and B). Correspondingly, TNF-induced protein expression was observed as indicated by a twofold increase in MMP3 levels from 5.5 ± 0.02 ng/mL to 10.5 ± 1.4 ng/mL (p ≤ 0.01), and a fivefold increase in IL8 protein from 111.4 ± 15.6 ng/mL to 512.2 ± 28.2 ng/mL (p ≤ 0.01, Figure 1C).

 As we aimed to investigate involvement of cPLA2α in regulating TNF-induced expression of these metabolites, we further characterized the PLA2G4A gene expression in synoviocytes. We found the PLA2G4A transcript to be expressed in untreated cells, and further induced 4.8 ± 1.1-fold by TNF (p ≤ 0.02, Figure 1D). Collectively, the SW982 synoviocyte model system was found suitable for investigating potential involvement of cPLA2α in regulating TNF-induced signaling related to joint destructive processes occurring in the RA synovium.

### AVX002 Efficiently Reduce AA Release and PGE2 Production

**Figure 2:**
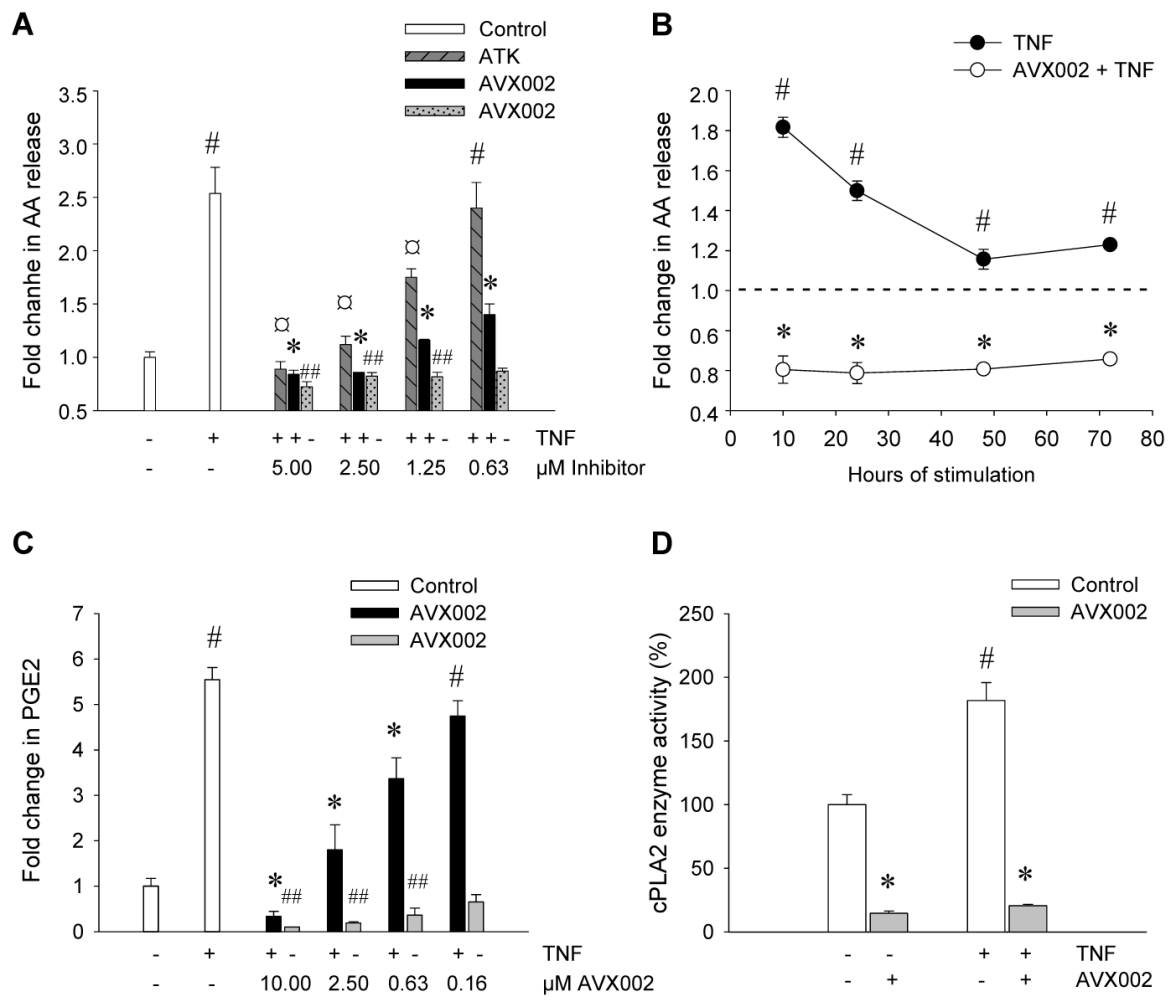
cPLA2α regulates TNF-induced AA release and PGE2 synthesis. SW982 synoviocytes were treated with either AVX002 or ATK (2 hrs) in indicated concentrations (A, C) or 5 µM (B) prior to stimulation with TNF (10 ng/mL) for 24 hrs (A, C) or indicated times (B). Analysis of released [^3^H]-AA (A, B) or PGE2 (C) was performed as described in the Methods section. Presented values are mean ± SD of TNF-induced release compared to untreated control samples in one representative of at least three independent experiments performed in duplicates (PGE2) or triplicates ([^3^H]-AA). AA release levels for untreated samples are indicated by a dash line in (B). Synoviocytes were stimulated with TNF (10 ng/mL) for 6 hours prior to lysis and detection of *in* *vitro* cPLA2α enzyme activity as described in the Methods section (D). Presented values are mean ± SD of *in* *vitro* cPLA2α activity (%) in one representative of at least three independent experiments performed in duplicates. Statistical significance is indicated as follows: A: ^#^p≤ 0.01 when compared to untreated control values, ^*^p ≤ 0.01 (AVX002) and ^¤^p ≤ 0.01 (ATK) when compared to TNF-stimulated control values. B: ^#^p ≤ 0.05 when compared to untreated control values, ^*^p ≤ 0.05 when compared to TNF-stimulated control values. C: ^#^p ≤ 0.01, ^##^p ≤ 0.02 when compared to untreated samples, and ^*^p ≤ 0.01 when compared to TNF-stimulated and control values. D: ^#^p ≤ 0.03 when compared to untreated cell lysates, ^*^p ≤ 0.02 when compared to untreated or TNF-stimulated cell lysates without inhibitor.

The AA metabolite PGE2 is recognized as a potent regulator of inflammation and the benefits of reducing pathological PGE2 levels are commonly accepted [12]. We aimed to investigate the effect of the recently described cPLA2α inhibitor AVX002 [25] on cellular AA release and total PGE2 synthesis in the synoviocyte model system. AVX002 dose-dependently inhibited the TNF-induced AA release with a mean estimated IC50 value of 0.9 ± 0.3 μM (Figure 2A). This estimate is based on 4 independent experiments. AVX002 alone modestly reduced basal AA release in a dose dependent manner; 5 µM AVX002 reduced basal AA release by 27%, 2.5 µM and 1.25 µM by 18%, and 0.63 µM by 13%. Furthermore, AVX002 was found to display long-lasting inhibitory effects evidenced by reduced TNF-induced AA release to 66 ± 2% of basal level following 72 hrs of TNF stimulation (p ≤ 0.05) (Figure 2B). AVX002 inhibitory efficacy was compared to the widely used commercial cPLA2α inhibitor, ATK [35]. ATK reduced TNF-induced AA release in a similar fashion as AVX002 (Figure 2A), however with significantly lower efficacy (p=0.01) as indicated by the higher mean estimated IC50 value of 2.9 ± 0.8 μM. 

Having shown that the inducing effects of TNF on AA release are normalized toward basal level by cPLA2α inhibitors, we next aimed to investigate if this finding was also reflected in the level of PGE2 production. By ELISA analysis, we demonstrated that production of PGE2 increased fivefold in response to TNF stimulation compared to basal level, from 52.6 ± 5.7 ng/mL to 265.5 ± 18.7 ng/mL (p ≤ 0.01, Figure 2C). TNF-induced PGE2 production was dose-dependently reduced by AVX002 with a mean estimated IC50 value of 1.3 ± 0.3 μM. The IC50 estimate is based on three independent experiments. AVX002 alone also significantly decreased PGE2 production in unstimulated cells in a dose-dependent manner by 64% in 0.63 μM, 80% in 2.5 μM and 90% in 10 μM concentrations, compared to untreated control samples. 

Since the metabolization of AA into PGE2 in response to TNF implies the involvement of the COX pathway, it was of interest to investigate the expression of the inducible COX2 enzyme. We show that in response to TNF, COX2 mRNA expression increased 9.0 ± 1.2-fold (p ≤ 0.01), an induction that was significantly reduced by 50% following AVX002 treatment (p ≤ 0.03, Figure 1D). Next, we investigated potential transcriptional regulation of the cPLA2α gene, PLA2G4A. We found that the fivefold induction by TNF was significantly reduced by 72% (p ≤ 0.03, Figure 1D), suggesting that cPLA2α activity may be subject to auto-regulation in response to TNF stimulation. AVX002 treatment alone did not significantly affect basal transcription of either gene (results not shown). Hence, AVX002 served to normalize, not short-circuit gene expression thereby still allowing basal house-hold activities. Taken together, the inhibitory effect of AVX002 was evident at several regulatory levels and at different points in time. cPLA2α and COX2 enzyme activities are normalized towards basal activity levels as monitored by means of AA release and PGE2 production and secretion. In parallel, COX2 and PLA2G4A gene expression are also reduced towards, but not below, basal levels. This suggests that the cPLA2α enzyme has an important regulatory role at several points in the AA cascade in synoviocytes.

### AVX002 inhibits cPLA2α activity in cell lysates

To validate the inhibitory effect of AVX002 on cPLA2α in our model system, we investigated the potency of AVX002 to restrain cPLA2α enzyme activity in cell lysates from untreated and TNF-stimulated cells. Lysates of TNF-stimulated cells displayed an 80% increase in activity of cPLA2α compared to untreated cells as indicated by increased hydrolysis and release of ^14^C-AA from phospatidylcholine visualized by TLC chromatography (Figure 2D). This stimulatory effect is consistent with the detected induction of AA release by TNF described above and validates the activation of cPLA2α in SW982 cells in response to TNF. Detection of cPLA2α activity in lysates from unstimulated cells indicate a basal enzyme activity in synoviocytes, as previously described in primary rheumatoid synoviocytes [36,37], and correlates to the basal release of AA detected in unstimulated cells (Figure 2A). Furthermore, AVX002 efficiently inhibited activity of cPLA2α in lysates from unstimulated cells, as well as TNF-stimulated cells (Figure 2D). The capacity of AVX002 in inhibiting cPLA2α enzyme activity in cell lysates agrees with the previously reported effect on recombinant cPLA2α [25]. Together, these results support our interpretation that cPLA2α is activated by TNF in synoviocytes, and that AVX002 directly inhibits cPLA2α activity resulting in reduced downstream AA release and PGE2 production. 

### MMP3 expression is regulated by AVX002

To evaluate a potential role for cPLA2α in regulating destruction of cartilage in RA, we analyzed the effects of AVX002 on MMP3 expression. Indeed, AVX002 dose-dependently reduced TNF-induced MMP3 transcription with a maximum inhibition of 69% at 5 μM (p ≤ 0.01, Figure 1A). Basal MMP3 mRNA expression was not significantly affected by AVX002 alone (results not shown). Moreover, the TNF-induced twofold increase in MMP3 protein secretion was significantly reduced by AVX002 by 68%, from 10.5 ± 1.4 ng/mL to 7.1 ± 0.5 ng/mL (p ≤ 0.01, Figure 1C). These results suggest a role of cPLA2α in regulating TNF-induced MMP3 expression in human synoviocytes.

### IL8 expression is regulated by AVX002

Next, the effect of AVX002 in regulating expression of the known neutrophil attractant IL8 was investigated by QPCR and ELISA immunoassay. AVX002 dose-dependently reduced TNF-induced up-regulation of IL8 transcription, with a maximum inhibition of 63% at 5 μM (p ≤ 0.03, Figure 1B). Basal IL8 gene expression was not affected by AVX002 alone (results not shown). AVX002 also reduced TNF-induced IL8 protein secretion significantly by 65%, from 512.2 ± 28.2 ng/mL to 252.7 ± 62.8 ng/mL (p ≤ 0.01, Figure 1C). Accordingly, our results suggest that in human synoviocytes, TNF-induced IL8 expression may be regulated by cPLA2α.

## Discussion

**Figure 3:**
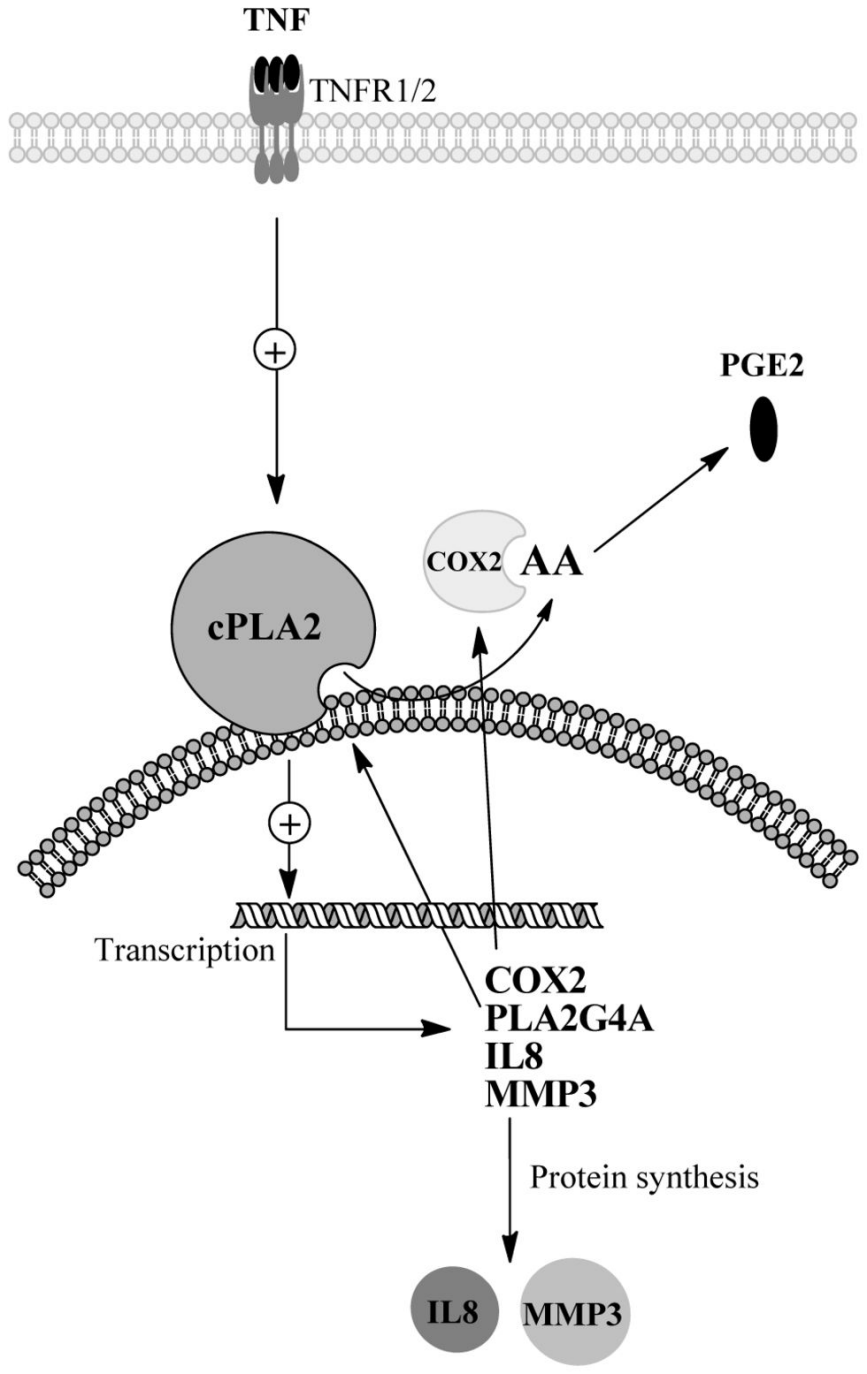
Proposed TNF-induced and cPLA2α dependent signaling in synoviocytes. TNF is a powerful inducer of inflammation and joint destruction in RA. In the SW982 synoviocyte model system, TNF induces activation of the cPLA2α enzyme to release AA from membranes, presumably through binding to its receptors TNF receptor 1 and TNF receptor 2 (TNFR1/TNFR2). AA released by cPLA2α is metabolized by COX2 to PGE2, a commonly recognized inducer of inflammation. The TNF-induced AA cascade can be self-reinforced by transcriptional regulation of COX2 and PLA2G4A genes. MMP3 and IL8 are central effectors in RA through cartilage destruction (MMP3), angiogenesis and attraction of immune cells (IL8). cPLA2α regulates TNF-induced expression of MMP3 and IL8 on transcriptional and protein levels. Consequently, cPLA2α functions to coordinate joint destructive and inflammatory processes in synoviocytes.

In RA, chronic inflammation and joint destruction is driven by excessive production of pro-inflammatory cytokines, chemokines and eicosanoids. In this study, by applying the chemical cPLA2α inhibitors AVX002 and ATK, we demonstrate that cPLA2α may be an important effector of TNF in intracellular signaling directly related to synovitis. Figure 3 summarizes the hypothesized involvement of cPLA2α in regulating synoviocyte expression of key mediators of bone and cartilage destruction, angiogenesis and recruitment of immune cells, along with the availability of AA and subsequent PGE2 production.

Our results demonstrate that AVX002 efficiently and persistently reduces AA-release and hence the availability of substrate for pro-inflammatory eicosanoid production in synoviocytes (Figure 2). We have previously demonstrated that AVX002 is a potent inhibitor of recombinant cPLA2α enzyme activity *in vitro* [25] and that AVX002 efficiently inhibits PGE2 production in IL-1β stimulated rat mesangial cells. Here, we provide proof of principle that AVX002 potently inhibits cPLA2α as we for the first time demonstrate that AVX002 is an inhibitor of cPLA2α enzyme activity in synoviocytes (Figure 2D). Analogous effects of cPLA2α inhibition of enzyme activity, cytokine-induced AA release, PGE2 production and gene expression has previously been demonstrated with three other chemical inhibitors resembling AVX002 and ATK in chemical structure; MAFP, and the trifluoromethyl ketone analogue of EPA (EPACOCF3) [38–41]. Based on these results and the herein reported observations on the effects of AVX002 and ATK in our model system, we postulate that the observed effects of AVX002 are due to inhibition of cPLA2α enzyme activity. 

The important role of PGE2 in propagating inflammation and pain is commonly recognized as reflected by the effective symptom relief of pain and stiffness by NSAID treatment in RA patients [12,13]. The highly pleiotropic PGE2 exhibits a wide range of biological actions [42] and is also proposed to be a central factor in bone and cartilage resorption in arthritis through regulation of osteoclast activity and expression of cytokines and MMPs in various model systems [43–45]. The versatile effects of PGE2 further include promotion of immune cell influx to inflamed tissue and angiogenesis [46,47]. As synoviocytes are important promoters of inflammation and joint destruction in RA, the reduced PGE2 production by AVX002 in these cells supports a key role for cPLA2α in RA pathogenesis. Our results further imply that reduced PGE2 synthesis in response to cPLA2α inhibitors may be self-reinforced through transcriptional regulation of the COX2 and PLA2G4A genes (Figure 1D). Transcriptional regulation of COX2 by cPLA2α has previously been shown in murine model systems [23,48] and our experiments suggest that this mechanism might be active also in human synoviocytes. The decrease in PLA2G4A mRNA in response to cPLA2α inhibition is to our knowledge a novel finding. It may suggest a feed-back loop from cPLA2α enzymatic activity through transcriptional regulation of the PLA2G4A gene in response to TNF in synoviocytes. Feed-back signaling where enzymatic activity of cPLA2α is required to regulate its own gene induction is previously reported in lung fibroblasts in response to IL-1β stimulation [9]. This possible auto-regulation in response to pro-inflammatory stimuli presents a potential self-reinforcing impact of inhibiting cPLA2α enzyme activity. 

As degradation of cartilage and bone are major hallmarks of RA, disruption of these destructive processes represents a central therapeutic objective. MMP3, with its wide range of substrate specificity and ability to activate other MMPs, is essential in RA cartilage degradation [30] and correlate with disease activity and inflammation markers in RA patients [49]. We identify cPLA2α as a possible regulator of MMP3 expression in human synoviocytes (Figure 1A and C). This correlates with reported findings from murine arthritis [23] suggesting that cPLA2α is a regulator of cartilage degradation in RA. Angiogenesis and the continuous influx of immune cells to the inflamed synovium are important processes driving the inflammation in RA joints. cPLA2α is proposed to be a regulator of neutrophil recruitment and inflammation in murine collagen-induced arthritis [22], and has been found to regulate expression of the chemotactic and angiogenic factor IL8 in human lung fibroblasts [50]. We show that cPLA2α enzyme activity might be involved in regulating IL8 production also in human synoviocytes (Figure 1B and C), emphasizing the potential biological relevance of cPLA2α in synovitis. 

Given the complexity of TNF signaling networks, focusing on an intracellular therapeutic target downstream the TNF receptor may show reduced adverse effects compared to TNF-blocking therapy as many key host defense mechanisms are not targeted. Accordingly, a modulation of cPLA2α enzyme activity by specific cPLA2α inhibitors and subsequently normalizing downstream signaling may represent an alternative or supplement to current therapeutic strategies for RA treatment. Indeed, cPLA2α is expressed in RA synovium [51], and has been shown to play an important role in inflammation and in several animal models of arthritis [20–22]. Furthermore, inhibitors of cPLA2α including ATK and pyrroxyphene ameliorate various inflammatory conditions including collagen-induced arthritis in mice [23,52]. Our results expand the understanding of cPLA2α as a possible regulator of inflammatory and joint destructive processes in human synoviocytes through regulation of MMP3, IL8 and PGE2. 

Taken together, we demonstrate that cPLA2α may have an important role in regulating TNF-induced intracellular signaling in synoviocytes. Hence, our results suggest that cPLA2α may be involved in both inflammatory, angiogenic and tissue destructive processes and may hence be a promising therapeutic target to reduce inflammation and discomfort, pain, reduced functionality and mobility associated with RA. 
